# University of Edinburgh

**Published:** 1846-08

**Authors:** 


*University of Edinburgh.*—Mr. John Goodsir, the well-known anatomist and microscopist, has been elected the Professor of Anatomy in this university.—*Ibid*

